# Anti-malarial drug quality in Lagos and Accra - a comparison of various quality assessments

**DOI:** 10.1186/1475-2875-9-157

**Published:** 2010-06-11

**Authors:** Roger Bate, Kimberly Hess

**Affiliations:** 1 American Enterprise Institute, Washington, D.C., USA; 2 Africa Fighting Malaria, Washington, D.C., USA

## Abstract

**Background:**

Two major cities in West Africa, Accra, the capital of Ghana, and Lagos, the largest city of Nigeria, have significant problems with substandard pharmaceuticals. Both have actively combated the problem in recent years, particularly by screening products on the market using the Global Pharma Health Fund e.V. Minilab^® ^protocol. Random sampling of medicines from the two cities at least twice over the past 30 months allows a tentative assessment of whether improvements in drug quality have occurred. Since intelligence provided by investigators indicates that some counterfeit producers may be adapting products to pass Minilab tests, the results are compared with those from a Raman spectrometer and discrepancies are discussed.

**Methods:**

Between mid-2007 and early-2010, samples of anti-malarial drugs were bought covertly from pharmacies in Lagos on three different occasions (October 2007, December 2008, February 2010), and from pharmacies in Accra on two different occasions (October 2007, February 2010). All samples were tested using the Minilab^® ^protocol, which includes disintegration and active ingredient assays as well as visual inspection, and most samples were also tested by Raman spectrometry.

**Results:**

In Lagos, the failure rate in the 2010 sampling fell to 29% of the 2007 finding using the Minilab^® ^protocol, 53% using Raman spectrometry, and 46% using visual inspection. In Accra, the failure rate in the 2010 sampling fell to 54% of the 2007 finding using the Minilab^® ^protocol, 72% using Raman spectrometry, and 90% using visual inspection.

**Conclusions:**

The evidence presented shows that drug quality is probably improving in both cities, especially Lagos, since major reductions of failure rates over time occur with all means of assessment. Many more samples failed when examined by Raman spectrometry than by Minilab^® ^protocol. The discrepancy is most likely caused by the two techniques measuring different aspects of the medication and hence the discrepancy may be the natural variation in these techniques. But other explanations are possible and are discussed.

## Background

### The dangers of substandard drugs

Substandard drugs can be lethal; diseases like malaria kill rapidly, and without effective drugs, death for a small percentage can come quickly. In addition to this problem, the Plasmodium parasite which causes malaria, adapts over time, becoming resistant to previously effective drugs. This adaptation is accelerated if the treatments are sub-strength - either low active ingredient, or low availability of active ingredient due to poor formulation or product degradation. It is crucial, therefore, that patients complete the treatment course and that their drugs are properly formulated. Sadly, in some areas where malaria is highly prevalent, treatments such as chloroquine, a cheap and safe drug, now fail to cure because parasites have developed resistance to it. Fake and substandard drugs that are under-dosed promote resistance. Combating such drugs is therefore important to ensure the continued survival of drugs to fight malaria.

### Nigeria and Ghana combat their fake drug problem

Since 2002, when nearly 41% of the drugs in the Nigerian market were estimated to be fake, Nigeria's National Agency for Food and Drug Administration and Control (NAFDAC) has made significant efforts in combating fake drugs [1,2]. NAFDAC has improved screening of drugs in the field; it has undertaken forensic analysis of low quality drugs and pursued those selling and marketing them. Screening has improved with the deployment of several small portable laboratories, known as Global Pharma Health Fund e.V. Minilabs^® ^for rapid product screening where formal laboratory facilities are sparse. NAFDAC is also conducting a survey and audit of all drugs on sale in order to build a pharmaceutical database. From 2002, drug failures fell to roughly 16% in 2006 and are now down to about 10%, according to the director-general of NAFDAC, Dr. Paul Orhii (personal communication, February 25, 2010). Dr. Orhii is pushing further by being the first anti-counterfeit department anywhere in the world to deploy six hand-held laser (Raman) spectrometers, which can provide immediate authentication of drugs. According to NAFDAC's Elizabeth Awagu, this deployment is helping to close down more of those locations still selling fake products.

As a participating country in the President's Malaria Initiative, Ghana has been assisted by the United States Pharmacopeia (USP) and others in improving diagnostics and laboratory facilities, including the deployment of Minilabs similar to NAFDAC's actions in Nigeria. In July 2009, one of the five sentinel sites set up under the guidance of USP, in association with Ghana's Food and Drugs Board Quality Control Laboratory, was rewarded when a local citizen brought in suspect artemether-lumefantrine (Coartem^®^) - a leading brand of the newest artemisinin-based combination therapy (ACT), which was found to contain no active ingredient. Tens of thousands of treatments of the fake were seized.

## Methods

Sampling methods deployed in the drug collections were developed in line with similar studies [3-5]. Anti-malarial drugs were obtained by local nationals from randomly selected private pharmacies in Lagos and Accra. Study agents posed as customers and were instructed to stay within a single neighbourhood and to select pharmacies at first sight on a random walk, and were blind as to the purpose for which they were collecting samples. They purchased a sample lot of anti-malarial tablet formulations, namely: sulphadoxine-pyrimethamine (SP), amodiaquine, mefloquine, artemisinin monotherapies and ACT. Agents were instructed not to purchase chloroquine since it is no longer an effectual treatment in nearly every country. All drugs in all pharmacies were available without a prescription. Treatment packs included drugs sold in the manufacturer's original packaging as well as those distributed loose, often in paper bags. Once purchased, all drugs were stored at ambient temperature, with low humidity and no sunlight, until testing.

All samples and packaging were visually inspected for obvious flaws in line with the protocol established by the Global Pharma Health Fund e.V. Minilab^®^. The Minilab was then used to run semi-quantitative thin-layer chromatography (TLC) and disintegration tests on each sample, within 60 days of collection, to determine the presence and relative concentration of active ingredients. Each test was run in duplicate, with the generous assumption that the result more consistent with the reference was recorded. The Minilab protocols award products a "pass" if they have 80% or more of the labeled active ingredient(s) (note there is no upper-bound limit). For fixed-dose combinations and SP, a "pass" was awarded only if both active ingredients met this standard. Quality control of the Minilab was performed daily prior to drug testing and consisted of performing TLC on Minilab-reference samples for the drug classes being analysed. In addition, Minilab reagents were quality control tested using reference samples when a new lot was introduced.

Samples were also tested using a portable Raman spectrometer (TruScan; Ahura Scientific, Inc., Wilmington, MA). Spectrometers "fingerprint" materials without using external substances. Unlike the Minilab assays which focus on specific attributes of the medicines, the spectra generated by the spectrometer reflect all contents of the sample: active pharmaceutical ingredients, excipients, fillers, dyes, and coatings. The spectra will change when any of these contents is changed or is inherently different due to different manufacturers producing drugs with different concentrations of excipients, and perhaps entirely different excipients. Furthermore, temperature degradation or moisture degradation of a sample will affect the spectra, which is critical when assessing the viability of compounds, such as artemisinin, whose effectiveness is lowered by moisture. Methods were established for the Raman spectrometer for each brand studied, and testing was carried out in the same location as the Minilab analysis. Of note, 39 samples could not be properly assessed because methods could not be established for those brands. For other brands, sample sizes were small, and while spectral methods were established for each brand, it is unknown if any reliability problems from such sampling occurred, potentially biasing results. Since the spectrometer is set to western compendial standards, methods should be assessed from a sizeable number of samples to avoid false negatives, which can be found with natural product variability. Unfortunately this was not possible with the majority of samples. Furthermore, unlike the Minilab assays, a failure by spectrometry may indicate an intellectual property violation of a brand, rather than a product which is a risk to public health.

### Sample collections

Between mid-2007 and early-2010, study agents conducted three samplings of pharmacies in Lagos (October 2007, December 2008, February 2010), and two samplings of pharmacies in Accra during the same period (October 2007, February 2010). A total of 339 samples were collected and tested using the Minilab, of which 300 samples were also tested with the same spectrometer (TruScan) deployed by the Nigerian Government. In all, 15 different pharmacies were sampled from the same area in Lagos; over the three collections, eight were sampled more than once due to the random selection protocol. Thirteen pharmacies were sampled in Accra, eight of which were sampled during both collections. It should be noted that sample sizes are not large; indeed, the 2007 sampling in Lagos included only 22 samples from only seven pharmacies.

Study agents were instructed to buy a variety of older anti-malarial therapies. These include therapies newer than chloroquine (not sought in this study but still available and a popular choice because of its low price and familiarity); but which have been on sale for most of the past thirty years - such as amodiaquine, mefloquine and SP. Study agents also bought artemisinin monotherapies where available. The World Health Organization (WHO) strongly recommends that artemisinin be combined with some other drug, which acts against malaria in a different way, as a multi-pronged attack will reduce the risk of the parasite developing resistance. It is disquieting that the single formulation, which the WHO has for over three years asked manufacturers not to produce or traders to sell, was still widely available [6].

At the time of the 2007 sampling, the only ACT on sale and testable using the Minilab was artemether-lumefantrine (AL); although Minilab testing of other forms of ACT became available after 2007, these were not sampled because of the testing constraint during the first collection. ACT is the first-line therapy recommended in every African nation (bar two small nations where chloroquine is still effective).

## Results

Tables 1, 2 and 3 summarize the findings of the drug testing results by drug type. Table 4 presents the failure rates recorded by visual inspection, Minilab tests, and Raman spectrometry tests for those drugs testable by spectrometry (300 of the original 339 samples - 39 samples of certain brands could not be assessed using the Raman spectrometer because original products were not available by manufacturers). At first glance, the reduction in failure rates identified by the Minilab in Accra and Lagos appears to be good news: the obvious implication, if the data reflect a wider phenomenon, is that anti-malarial drug quality is improving in these cities. The overall failure rate for Minilab tests (TLC assay and/or disintegration) drops significantly from the 2007 sampling to the 2010 sampling in Lagos (35% or 7/20 to 10.3% or 9/87), and in Accra (35.3% or 12/34 to 19.1% or 9/47). Packages were visually screened for apparent trademark violations or other obvious labeling infractions, which might imply the product was counterfeit, or where the packaging was so poor that the product was obviously degraded. Products were treated as suspicious if, for example, the manufacturing dates were after the product expiry dates, there were incorrect spellings of product name or details of production, or there were odd fonts or font sizes different from innovator brands and branded generic originals. There was a slight improvement in the visual inspection failure rates from the 2007 sampling to the 2010 sampling: from 15% (3/20) to 6.9% (6/87) in Lagos and 11.8% (4/34) to 10.6% (5/47) in Accra.

**Table 1:** Drug samples failing Minilab tests (API assay and disintegration)^a^

Sampling	Older **monotherapies**^b^	Artemisinin monotherapies	**ACTs**^**c**^	Total Failures
Lagos 2007	3/8	3/7	1/7	7/22

Lagos 2008	8/59	12/65	1/5	21/129

Lagos 2010	5/30	6/47	1/17	12/94

**Lagos Total**	**16/97**	**21/119**	**3/29**	**40/245**

Accra 2007	5/13	5/16	3/8	13/37

Accra 2010	5/23	5/24	1/10	11/57

**Accra Total**	**10/36**	**10/40**	**4/18**	**24/94**

**TOTAL**	**26/133**	**31/159**	**7/47**	**64/339**

a. Numbers are total that failed testing/total samples tested

b. Includes amodiaquine, mefloquine or sulphadoxine-pyrimethamine

c. Artemisinin-based combination therapies

**Table 2:** Drug samples failing visual inspection tests^a^

Sampling	Older **monotherapies**^b^	Artemisinin monotherapies	**ACTs**^**c**^	Total Failures
Lagos 2007	1/8	1/7	1/7	3/22

Lagos 2008	3/59	9/65	0/5	12/129

Lagos 2010	4/30	3/47	0/17	7/94

**Lagos Total**	**8/97**	**13/119**	**1/29**	**22/245**

Accra 2007	1/13	2/16	1/8	4/37

Accra 2010	2/23	4/24	0/10	6/57

**Accra Total**	**3/36**	**6/40**	**1/18**	**10/94**

**TOTAL**	**11/133**	**19/159**	**2/47**	**32/339**

a. Numbers are total that failed testing/total samples tested

b. Includes amodiaquine, mefloquine or sulphadoxine-pyrimethamine

c. Artemisinin-based combination therapies

**Table 3:** Drug samples failing Raman spectrometry tests^a^

Sampling	Older monotherapies^b^	Artemisinin monotherapies	**ACT**^**c**^	**Total Failures**^**d**^
Lagos 2007	3/6	3/7	1/7	7/20

Lagos 2008	10/49	17/58	1/5	28/112

Lagos 2010	8/28	7/44	1/15	16/87

**Lagos Total**	**21/83**	**27/109**	**3/27**	**51/219**

Accra 2007	5/11	6/15	3/8	14/34

Accra 2010	5/16	8/23	1/8	14/47

**Accra Total**	**10/27**	**14/38**	**4/16**	**28/81**

**TOTAL**	**31/110**	**41/147**	**7/43**	**79/300**

a. Numbers are total that failed testing/total samples tested

b. Includes amodiaquine, mefloquine or sulphadoxine-pyrimethamine

c. Artemisinin-based combination therapy

d. Only 88% of total samples were tested by spectrometry (300 out of 339), since the TruScan spectrometer requires a known good quality version for comparison and for 39 samples this could not be established.

**Table 4:** Drug samples (in testable form for spectrometry analysis) failing quality tests^a^

Sampling	Total Minilab failures	Total visual inspection failures	Total Raman spectrometry failures
Lagos 2007	(7/20) 35%	(3/20) 15%	(7/20) 35%

Lagos 2008	(18/112) 16.1%	(11/112) 9.8%	(28/112) 25%

Lagos 2010	(9/87) 10.3%	(6/87) 6.9%	(16/87) 18.4%

**Lagos Total**	**(34/219) 15.5%**	**(20/219) 9.1%**	**(51/219) 23.3%**

Accra 2007	(12/34) 35.3%	(4/34) 11.8%	(14/34) 41.2%

Accra 2010	(9/47) 19.1%	(5/47) 10.6%	(14/47) 29.8%

**Accra Total**	**(21/81) 25.9%**	**(9/81) 11.1%**	**(28/81) 34.6%**

**TOTAL**	**(55/300) 18.3%**	**(29/300) 9.7%**	**(79/300) 26.3%**

a. Percentages are supported by (total that failed testing/total samples tested)

This trend is continued with the drugs that failed Raman spectrometer testing, although the spectrometer fails a higher number of samples than visual inspection or the Minilab assays. A similar finding was made in the comparison of Minilab failure rates, which were far lower than with the more sophisticated laboratory compendia assessments, in the study of the Quality of Antimalarials in sub-Saharan Africa (QAMSA). This is the product of the Promoting the Quality of Medicines (PQM) partnership funded by USAID and implemented by USP. The first results, from Madagascar, Uganda and Senegal, are part of a larger, ten-country study (QAMSA) implemented by WHO in collaboration with USP.

Table 4 provides an interesting assessment of the improvement in drug quality. For Lagos, samples failing Minilab tests in the 2010 sampling fell to 29% (10.3/35 *100) of the 2007 sampling; those failing visual inspection fell to 46% (6.9/15 *100) of the 2007 sampling, and those failing spectrometry fell to 53% (18.4/35 *100). For Accra, the respective figures are 54% (19.1/35.3 *100), 90% (10.6/11.8 *100) and 72% (29.8/41.2 *100). From the 2008 sampling to the 2010 sampling in Lagos, the fall is to 64% (10.3/16.1 *100) for Minilab and 74% (18.4/25 *100) for spectrometry.

## Discussion

Since all chosen drug quality assessment methods show a reduction in failure rates, it is reasonable to conclude that the quality of anti-malarial drugs in the two cities has probably improved between October 2007 and February 2010. The data are not sufficient to draw any firm conclusions about differences between spectrometry and Minilab results, particularly because the smallest differences occur with the largest samples. Assuming the data reflect real changes in the cities' drug markets, what might they mean?

It has been known for at least five years that the Nigerian authorities, NAFDAC, and more recently, the Ghanaian authorities, have been undertaking serious anti-counterfeiting efforts. It is therefore quite likely that at least some counterfeiters and those wilfully supplying substandard products, which involve no labeling infractions, have left the market in search of less well-regulated locations. A more speculative possibility is that some of these actors may have adapted in order to bypass or overcome such efforts. This speculation has some weak support.

Ignoring visual inspection failures, which are a predictor of quality and a useful guide for inspectors but are not a quality assessment, there is a potentially important relative disparity between spectrometry testing and Minilab testing results. Assuming an overall improvement in drug quality, one would expect the failure rate to fall by roughly the same relative amount in both methods of assessment. Yet, for the same drugs there is a notably lesser quality improvement shown by spectrometry testing than the Minilab from the 2010 testing as compared with the 2007 testing. The discrepancy is 24% for Lagos (53% spectrometry, 29% Minilab) and 18% for Accra (72% spectrometry, 54% Minilab). For the 2008 testing to the 2010 testing in Lagos the difference is only 10% (74% spectrometry, 64% Minilab), and this may be the most significant result because the 2008 sampling in Nigeria was the largest by volume.

Anecdotal evidence from other markets suggests that counterfeiters have learned to include small amounts of active pharmaceutical ingredient (API) to pass basic analysis, such as colour dye tests, which tests only for the presence of API, not the quantity. The Minilab resolves this problem since its assay assesses semi-quantitatively the concentration of API. However, from conversations with investigators in India and China (probably the two nations producing the most fake and substandard products), more counterfeiters are adding roughly the right amount of API. This does increase the cost of production and if API is hard to get, such as in the case of artemisinin, this may increase the likelihood of exposure and hence capture. Nevertheless, according to investigators, such as Indian specialist, Suresh Sati, buying more API is still a relatively low cost part of the operation (packaging costs and infiltrating legitimate supply chains is where investigators claim most of the money is spent).

After all, with greater API there is still no guarantee that producers will formulate drugs properly, since it requires real production skill to make a bioequivalent product, but such counterfeit products might possibly fool the Minilab API assay. Given the scarcity of national laboratories to inspect products, which may be borderline Minilab API assay failures, such a response by counterfeiters may mean they escape detection - even in better-protected locations like Nigeria. It may be that the newer, but still substandard products have changed, partly in response to better detection, possibly worsening the problem of drug resistance. Such drugs may have positive impact on the patients' condition, but are more likely not to cure, and are more likely to promote resistance, and thus pose a much more insidious risk.

Of course, a happier and perhaps more likely interpretation is that work being done by the governments of Ghana and Nigeria, the legitimate pharmaceutical supply chain and others, such as USP, is showing success, and the discrepancy between the results of the spectrometry and Minilab tests is a statistical outlier. Or it is perhaps explained by some unreliable methods established for the spectrometer given the limited reference samples, which could increase the chance of false negatives.

## Conclusions

The data presented in this paper show a marked improvement in anti-malarial drug quality in Accra and especially Lagos over the past three years, as indicated by all of the quality tests undertaken. It is to be hoped that this reflects a general improvement in quality in these two cities rather than some statistical anomaly.

Many more samples failed when examined by Raman spectrometry than by Minilab^® ^protocol. The discrepancy is most likely caused by the two techniques measuring different aspects of the medication and hence the discrepancy may be the natural variation in these techniques. But other explanations are possible. Since one possible explanation, which has some anecdotal support, is that counterfeiters are adapting their actions to fool the more basic Minilab API assay, while not making a quality product, it is recommended that all users of the Minilab API assay periodically test some of their samples, which pass the API assay, with other techniques.

## Competing interests

The authors declare that they have no competing interests.

## Authors' contributions

RB designed and carried out all experiments and prepared the first draft of the manuscript. KH assisted with data analysis and editing of the manuscript. Both authors read and approved the final manuscript.

## References

[B1] Ebeleke E NAFDAC destroys N320 m fake products Vanguard 2010 http://www.vanguardngr.com/2010/01/28/nafdac-destroys-n320m-fake-products/

[B2] Taylor RB Shakoor O Behrens R Everard M Low A Wangboonskul J Reid R Kolawole J Pharmacopoeial quality of drugs supplied by Nigerian pharmacies Lancet 2001 357 1933 1936 10.1016/S0140-6736(00)05065-0 11425415

[B3] Bate R Coticelli P Tren R Attaran A Antimalarial drug quality in the most severely malarious parts of Africa - A six country study PLoS ONE 2008 3 e2132 doi:10.1371/journal.pone.0002132. 10.1371/journal.pone.0002132 18461128 PMC2324203

[B4] Bate R Tren R Hess K Attaran A Physical and chemical stability of expired fixed dose combination artemether-lumefantrine in uncontrolled tropical conditions Malar J 2009 8 33 2875-8-33 doi:10.1186/1475 10.1186/1475-2875-8-33 19243589 PMC2649943

[B5] Bate R Tren R Hess K Mooney L Porter K Pilot study comparing technologies to test for substandard drugs in field settings African Journal of Pharmacy and Pharmacology 2009 3 165 70

[B6] World Health Organization WHO calls for an immediate halt to provision of single-drug artemisinin malaria pills http://www.who.int/mediacentre/news/releases/2006/pr02/en/index.html 16736569

